# Giving Women What They Want: Reporting Breast Arterial Calcification on Mammograms at Northwell Health System

**DOI:** 10.1016/j.jacadv.2025.101914

**Published:** 2025-06-19

**Authors:** Nisha I. Parikh, Jean M. Cacciabaudo, Varinder P. Singh, Nina S. Vincoff

**Affiliations:** a Northwell, New Hyde Park, New York, USA; b Cardiovascular Institute, Northwell Health, New Hyde Park, New York, USA; c Katz Institute of Women’s Health, Northwell Health, New Hyde Park, New York, USA; d Division of Breast Imaging, Department of Radiology, Northwell Health, New Hyde Park, New York, USA

**Keywords:** breast arterial calcification, prevention, screening, subclinical atherosclerosis, women

Beginning in 2024, across our large Northwell Health System, we began universal reporting of breast arterial calcification (BAC) on all mammograms. Our choice to do this was largely driven by patient preference to know their BAC results and the growing scientific evidence showing that the presence of BAC both correlates with cardiometabolic risk factors and predicts later cardiovascular disease (CVD) risk in women. As Northwell clinical leaders in women’s health, CVD and breast radiology, we describe our rationale for this decision, our approach to treating the patient with BAC detected on mammography. We call for other health systems to consider BAC reporting on their mammograms.

## Brief background and history of BAC

As recently described by Daniels and Itchhaporia^1^ in *JACC: Advances*, BAC is medial arterial calcification that can be detected and quantified on a mammogram and its presence is associated with an increased CVD risk in women. BAC rises with age; the prevalence is zero in a 20-year-old person and as high as 50% at age 80 years.^2^ BAC has been demonstrated to have a relatively high correlation with coronary artery calcification (CAC), although prior studies may have limited generalizability due to the use of highly selected populations; indeed, prior investigations have relied on data from patients who have had mammograms and concurrent CAC scans for any other indication rather than on an unselected cohort of patient with prospectively measured CAC. Therefore, we believe our BAC patient registry can also serve as a powerful tool to study remaining unanswered questions regarding the utility of BAC in predicting and preventing later CVDs in women.

## BAC as a form of incidental arterial calcification

Arterial calcification in anatomical beds other than the breast has been reported in radiologic studies because both patients and providers have expressed a desire to know about these findings. As an example, CAC seen on noncardiac chest computed tomography scans are not only reported but also often quantified at many medical centers (in some cases with an Agatston score). Furthermore, aortic atheroma is reported routinely on transesophageal echocardiograms and when present will prompt us to counsel our patients to do intensive risk factor modification. It is worth noting that CAC is probably one of the better studied forms of arterial calcification. Indeed, CAC has a strong association with later cardiac disease events and is largely considered to be “equivalent” to having subclinical atherosclerosis. Although BAC is of increasing interest in research, the association of BAC with CVD outcomes is less well studied when compared with CAC with cardiovascular outcomes. Our Northwell BAC registry may help provide missing evidence regarding the relationship between BAC, subclinical atherosclerosis, and CVD outcomes.

## Patient preference and ease of reporting

Currently, there are no U.S. or international formal guidelines for including BAC on mammogram reports, and multiple studies have demonstrated that BAC is not consistently reported by radiologists.^3^, ^4^, ^5^, ^6^ Recent studies indicate that both patients and physicians prefer to be informed about the presence of BAC.^7^^,^^8^

In a 2023 study at Northwell Health, we demonstrated the potential of BAC notification to promote preventive cardiovascular health.^9^ Nearly 500 women were directly notified about the presence or absence of BAC on their mammogram and patients with positive BAC were surveyed at 3 months. Fifty-seven percent of survey respondents reported discussing their BAC results with a primary care physician or cardiologist. Seventy-six percent agreed that all women should be informed about BAC on their mammogram. These results prompted Northwell Health to begin universal reporting of BAC on mammography in early 2024. From January through October 2024, Northwell has reported BAC results on 87,907 mammogram reports and has identified BAC on 11,807 mammograms (13.4%). In the first year of this program, we expect to report BAC on approximately 100,000 mammograms and detect >13,000 positive cases. The additional time it takes for our breast radiologists to report the presence of BAC is minimal and has not materially added to mammogram reading time. Given the strong patient preference and the ease of accurate BAC reporting, we elected to universally report BAC on all Northwell Health mammograms.

## Clinical pathways for patients with BAC in cardiovascular prevention

**Figure 1:**
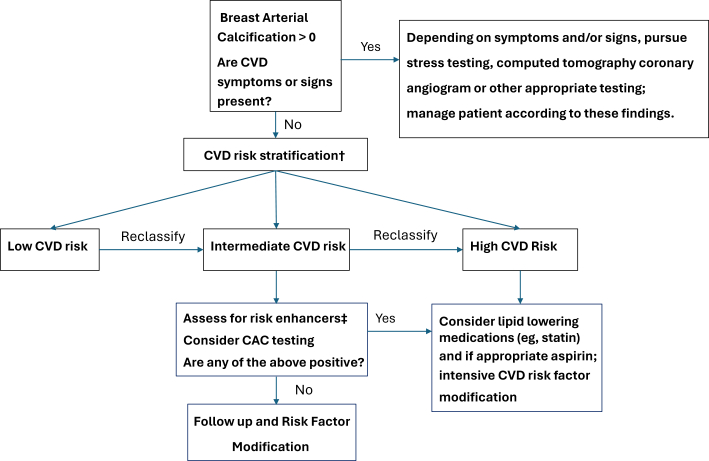
Patient With BAC: Northwell Women's Heart Program Clinical Care Algorithm∗ ∗If patient does not have a primary care provider or prefers, one can refer to Northwell Women’s Heart Program † based on CVD risk calculator, for example, PREVENT, SCORE^2^, etc. ‡Risk enhancers include: clinical history factors such as adverse pregnancy outcomes, rheumatologic diseases, psychosocial factors, hsCRP, Lp(a), ABI, cIMT, etc. This is our Northwell Women’s Heart Program clinical care algorithm for cardiovascular disease screening and risk stratification for the patient who has an incidentally noted BAC on mammogram. ABI = ankle brachial index; BAC = breast arterial calcification; cIMT = carotid intima media thickness; CVD = cardiovascular disease; CAC = coronary artery calcification; hsCRP = high-sensitivity C-reactive protein; Lp(a) = lipoprotein(a); PREVENT Risk score = Preventing Risk of Cardiovascular Disease Events; SCORE2 = Systematic Coronary Risk Evaluation 2.

With the universal reporting of BAC in our health system, our Northwell Women’s Heart Program elected to use the presence of BAC as a starting point for a cardiovascular screening and preventive visits. We use this information to engage in shared decision-making with women about the use of more intensive modification of CVD risk factors including lifestyle changes and statin therapy when warranted. We view the mammogram findings as a starting point for preventive care in many of our patients. For instance, in a 50-year-old patient who is intermediate risk for CVD and has a mammogram findings of BAC, we may choose to gather additional biomarkers to evaluate inflammatory risk information such as high-sensitivity C-reactive protein, lipoprotein(a), and a coronary artery calcium scan that can further aid in risk stratification (see Figure 1). We are often able to refine CVD risk in patients, particularly in low- or intermediate-risk patients. The results of these tests may help guide shared decision-making about the initiation and intensity of lifestyle modification and pharmacotherapy such as lipid-lowering therapy. What is less certain is the significance of a BAC of zero. One small study suggested it could rule out more severe CAC or ≥300 AU in women <55 years of age.^10^

## Future directions and call to action

Although there are many unanswered questions with respect to the ideal clinical pathways for women with BAC seen on mammography, we have taken the step to universally report BAC on our mammograms at Northwell Health and we believe that other health systems consider doing the same. We base this on the weight of evidence pointing toward an association between BAC and later CVD in women and a very strong patient and provider preference for knowing these results. We welcome the ability to study and refine ideal clinical implementation pathways within our large, diverse patient population. We anticipate our Northwell BAC patient registry will fill several existing gaps in scientific evidence. Finally, we anticipate that universal BAC reporting will help pave the way for mammograms to become a powerful screening and prevention tools for 2 of the most important women’s health concerns: breast cancer and CVD.

## Funding support and author disclosures

The authors have reported that they have no relationships relevant to the contents of this paper to disclose.
